# Transcriptional Profiling of STAT1 Gain-of-Function Reveals Common and Mutation-Specific Fingerprints

**DOI:** 10.3389/fimmu.2021.632997

**Published:** 2021-02-17

**Authors:** Simone Giovannozzi, Jonas Demeulemeester, Rik Schrijvers, Rik Gijsbers

**Affiliations:** ^1^Allergy and Clinical Immunology Research Group, Department of Microbiology, Immunology and Transplantation, KU Leuven, Leuven, Belgium; ^2^Laboratory for Viral Vector Technology and Gene Therapy, Department of Pharmaceutical and Pharmacological Sciences, KU Leuven, Leuven, Belgium; ^3^Laboratories for Computational Biology and Reproductive Genomics, Department of Human Genetics, KU Leuven, Leuven, Belgium; ^4^Cancer Genomics Laboratory, The Francis Crick Institute, London, United Kingdom; ^5^Leuven Viral Vector Core, KU Leuven, Leuven, Belgium

**Keywords:** STAT1, gain of function, mRNA sequencing, transcriptomic analysis, hypermorphic mutation, interferon gamma, gas

## Abstract

STAT1 gain-of-function (GOF) is a primary immunodeficiency typically characterized by chronic mucocutaneous candidiasis (CMC), recurrent respiratory infections, and autoimmunity. Less commonly, also immunodysregulation polyendocrinopathy enteropathy X-linked (IPEX)-like syndromes with CMC, and combined immunodeficiency without CMC have been described. Recently, our group and others have shown that different mutation-specific mechanisms underlie STAT1 GOF *in vitro*, including faster nuclear accumulation (R274W), and reduced mobility (R321, N574I) to near immobility in the nucleus (T419R) upon IFNγ stimulation. In this work, we evaluated the transcriptomic fingerprint of the aforementioned STAT1 GOF mutants (R274W, R321S, T419R, and N574I) relative to STAT1 wild-type upon IFNγ stimulation in an otherwise isogenic cell model. The majority of genes up-regulated in wild-type STAT1 cells were significantly more up-regulated in cells expressing GOF mutants, except for T419R. In addition to the common interferon regulated genes (IRG), STAT1 GOF mutants up-regulated an additional set of genes, that were in part shared with other GOF mutants or mutation-specific. Overall, R274W and R321S transcriptomes clustered with STAT1 WT, while T419R and N574I had a more distinct fingerprint. We observed reduced frequency of canonical IFNγ activation site (GAS) sequences in promoters of genes up-regulated by all the STAT1 GOF mutants, suggesting loss of DNA binding specificity for the canonical GAS consensus. Interestingly, the T419R mutation, expected to directly increase the affinity for DNA, showed the most pronounced effects on the transcriptome. T419R STAT1 dysregulated more non-IRG than the other GOF mutants and fewer GAS or degenerate GAS promotor sequences could be found in the promoter regions of these genes. In conclusion, our work confirms hyperactivation of common sets of IFNγ-induced genes in STAT1 GOF with additional dysregulation of mutation-specific genes, in line with the earlier observed mutation-specific mechanisms. Binding to more degenerate GAS sequences is proposed as a mechanism toward transcriptional dysregulation in R274W, R321S, and N574I. For T419R, an increased interaction with the DNA is suggested to result in a broader and less GAS-specific response. Our work indicates that multiple routes leading to STAT1 GOF are associated with common and private transcriptomic fingerprints, which may contribute to the phenotypic variation observed *in vivo*.

## Introduction

Signal Transducer and Activator of Transcription 1 (STAT1) gain-of-function (GOF) is a monogenic autosomal dominant (AD) disorder first described in 2011 (1, 2). To date 105 different mutations in at least 400 patients (3) have been reported, all presenting with increased STAT1-dependent cellular responses and high levels of tyrosine phosphorylated STAT1 (pSTAT1) in different immune cells. While the common phenotypic denominator is chronic mucocutaneous candidiasis [CMC—in 98% of the patients (4)], less frequent phenotypes were also associated with STAT1 GOF, like John Cunningham (JC)-virus induced progressive multifocal leukoencephalopathy (5), immunodysregulation polyendocrinopathy enteropathy X-linked (IPEX)-like syndromes with CMC (6, 7), Orf infection (8), and combined immunodeficiency (CID) without CMC (9). Currently, treatment is symptomatic, and consists of chronic antifungal therapy for CMC and episodic antibiotics as well as immunosuppressive treatment in case of auto-immune manifestations. In addition, hematopoietic stem cell transplantation and use of jakinibs has been reported in a minority of patients with varying success (10).

STAT1 is a transcription factor of the STAT family, that plays a key role in the immune response and the Interferon (IFN) signaling pathway, modulating diverse cellular processes including proliferation, cell death and cell differentiation (11–13). In unstimulated conditions STAT1 is located in the cytoplasm as an antiparallel homodimer (14, 15). Following IFNγ stimulation, STAT1 is phosphorylated at Y701 (11) and the antiparallel homodimers change their conformation to parallel homodimers (16, 17), that in turn are imported into the nucleus to bind gamma interferon (IFNγ) activation site (GAS) sequences in promoter regions and, up- or down-regulate the transcription of interferon regulated genes (IRGs) (18–20).

**Figure 1:**
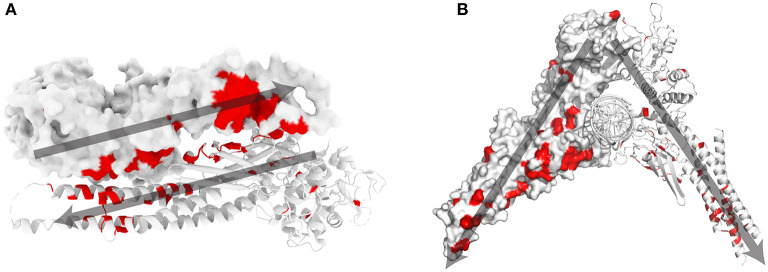
STAT1 crystal structure and known GOF mutations (3). **(A)** Schematic representation of antiparallel STAT1 homodimer (PDB reference 1yvl). **(B)** Parallel STAT1 homodimer bound to DNA (PDB reference 1bf5). For clarity, one monomer is represented in surface mode, the other as ribbons. In red amino acids for which GOF mutations have been shown. Dark gray arrows indicate the orientation of each monomer in the respective dimer configurations.

Most STAT1 GOF mutations [48 out of 74 amino acid (aa) positions] are located at the antiparallel homodimer interface (Figures 1A,B). A small subset of mutations are located in close proximity to the parallel homodimer-DNA interface (M325, H328, Q330, S466, and T419), while 18 mutated aa were described in positions away from the antiparallel homodimer and parallel homodimer-DNA interfaces.

Recently, we and others (21–24) described that specific STAT1 GOF mutations are associated with different molecular mechanisms. We studied a subset of STAT1 GOF mutants (R274W, R321S, T419R, and N574I) that reside in different domains and at different interfaces of the STAT1 protein (24). At least three different mechanisms could be identified: (I) faster nuclear import was observed for the R274W mutation (antiparallel homodimer interface); (II) reduced nuclear mobility and delayed dephosphorylation for the R321S and the N574I mutants (distant from the homodimer or DNA interfaces); (III) slower diffusion in the nucleus, possibly due to enhanced affinity for chromatin, was observed for the T419R mutation (parallel homodimer-DNA interface).

Exploring the crystal structures of parallel and antiparallel STAT1 dimers provides hints, at least for some mutations, on the underlying molecular mechanism. For example, R274 is located at the interface of the antiparallel homodimer, in a coiled-coil domain. We hypothesized that mutating the positively charged aa R274 may destabilize the antiparallel, inactive, homodimer, resulting in a faster shift to the active parallel form (24). Alternatively, aa T419 is in close contact with the DNA backbone when STAT1 is in its active parallel conformation. Mutating threonine to a positively charged arginine (T419R GOF mutation), may increase the affinity of STAT1 for the negatively charged DNA backbone.

Interestingly, aa N574 coordinates K511 of the same monomer in both the parallel and antiparallel conformation of the homodimer via a polar contact. In addition, a recent study demonstrated that STAT1 K511 is ubiquitinated, and that K511R mutation abolished ubiquitination and resulted in enhanced transcription of Type I interferon regulated genes (one Type II interferon regulated gene, *Ifit1*, was also analyzed, but its expression was not affected) (25). Due to the close proximity and direct interaction between N574 and K511, the N574I GOF mutation might affect STAT1 ubiquitination, which in turn could result in an up-regulation of IRG (24).

While the transcriptomic fingerprints of R274Q (21) and two other GOF mutants (R274G and H629Y) were studied individually (26), a comparison between the transcriptome of different STAT1 GOF mutations and their underlying molecular mechanisms is lacking. Therefore, we employed the STAT1^−/−^ U3A cell model and complemented these cells with the respective STAT1 GOF mutations using stable integration via lentiviral vector technology. This isogenic setup limited variability and to allowed direct comparison of the transcriptome following IFNγ stimulation. As controls, we included non-transduced STAT1^−/−^ U3A cells (NT) and cells complemented with STAT1 WT.

## Methods

### Cell Line Generation and Culture

U3A STAT1^−/−^ cells (Sigma, REF 12021503-1VL) were stably complemented using lentiviral vectors carrying STAT1-GFP WT, R274W, R321S, T419R, or N574I as previously described (24). The mutants R274W, R321S, and N574I were initially chosen in the context of our research hypothesis, because they are present in different domains of the STAT1 protein and because patients bearing these mutations were in follow-up at our university hospital. The T419R mutant was additionally included as a clinical GOF mutation predicted to directly interact with the STAT1 target DNA (24). All cells were cultured at 37°C (humidified atmosphere, 5% CO_2_) in DMEM (GIBCO-BRL) supplemented with 10% FBS (GIBCO, REF 10270-106), 0.01% v/v gentamicin (GIBCO-BRL). Cells were tested to be mycoplasma-free by PlasmoTest™, InvivoGen Europe.

### IFNγ Stimulation and mRNA Extraction

Five replicates of each cell line were independently seeded (500.000 cells/well). After 24 h the cells were either stimulated, by substituting the medium with DMEM 10% FBS, supplemented with IFNγ 1U/μl (Roche, REF 11040596001) for 4 h, or left unstimulated. Total mRNA was isolated using Aurum^TM^ total RNA mini kit (Biorad, REF 7326820). The 4 h time-point after IFNγ stimulation, was chosen because, (i) we showed that 4 h IFNγ stimulation is sufficient to upregulate ISGs (24) and (ii) to limit transcriptome effects that may result from activation of secondary pathways and later transcriptome effects.

### mRNA Library Preparation and Sequencing

RNA samples were processed by the Genomics Core Leuven (Belgium). Sequence libraries were prepared with the Lexogen QuantSeq 3′ mRNA-Seq Library prep kit according to the manufacturer's protocol. Samples were indexed to allow for multiplexing. Library quality and size range was assessed using a Bioanalyzer (Agilent Technologies) with the DNA 1000 kit (Agilent Technologies, California, USA) according to the manufacturer's recommendations. Libraries were diluted to a final concentration of 2 nM and subsequently sequenced on an Illumina HiSeq4000 platform. Single-end reads of 50 bp length were produced with a minimum of 1.5 M reads per sample. Quality control of raw reads was performed with FastQC (27). FastQC: a quality control tool for high throughput sequence data. (Available online at: http://www.bioinformatics.babraham.ac.uk/projects/fastqc) Adapters were filtered with ea-utils fastq-mcf (28) (https://github.com/ExpressionAnalysis/ea-utils). Splice-aware alignment was performed with STAR v2 (29) against the human reference genome hg37 using the default parameters. Reads mapping to multiple loci in the reference genome were discarded. Resulting BAM alignment files were handled with Samtools v1.5 (30). Quantification of reads per gene was performed with HT-Seq Count v2.7.14.

### Differential Expression Analysis

Differential expression analysis was performed with R-based (The R Foundation for Statistical Computing, Vienna, Austria) Bioconductor package DESeq2 (31). Reported *p-*values were adjusted for multiple testing with the Benjamini-Hochberg procedure, which controls false discovery rate (FDR). Genes that showed at least a 2-fold difference between the stimulated and the unstimulated condition were included in subsequent analyses. Principal component analysis (PCA) was performed using the pcaExplorer package (32).

### IRGs and Promoter Analysis

Interferon regulated genes (IRGs) are genes up- or down-regulated by the interferon signaling. The analysis of IRGs was performed using the Interferome database (http://www.interferome.org), a manually curated database of type I, II and III interferon-regulated genes. In our analysis we focused on type II IRGs. Promoter regions were obtained from the Eukaryotic Promoter Database (https://epd.epfl.ch/). Occurrence profile analysis and Position Weight Matrix scanning were performed on the Signal Search Analysis Server (https://ccg.epfl.ch/ssa/) (33). HOCOMOCO STAT1 consensus was used as definition of canonical GAS sequence (34). ChIP-Seq data were retrieved from the GEO database (GSE15353) (35) and correlation with promoters of genes of interest was evaluated using the ChIP-Seq online analysis tool from the Swiss Institute of bioinformatics (https://ccg.epfl.ch/chipseq/) (36).

### Pathways Analysis

Pathway enrichment analysis was performed using Ingenuity Pathways Analysis (IPA, http://www.ingenuity.com/products/ipa), using a cut-off of ±1 log_2_-fold change compared to the unstimulated condition and *p* < 0.001 for gene selection. Pathway activation is scored using gene expression z-scores. Briefly, a Z-score is defined as the difference between the error-weighted mean of the expression values of the genes in each pathway and the error-weighted mean of all genes in a sample after normalization. Z-scores were computed and plotted in a matrix of pathway activation scores.

### Statistics

**Figure 2:**
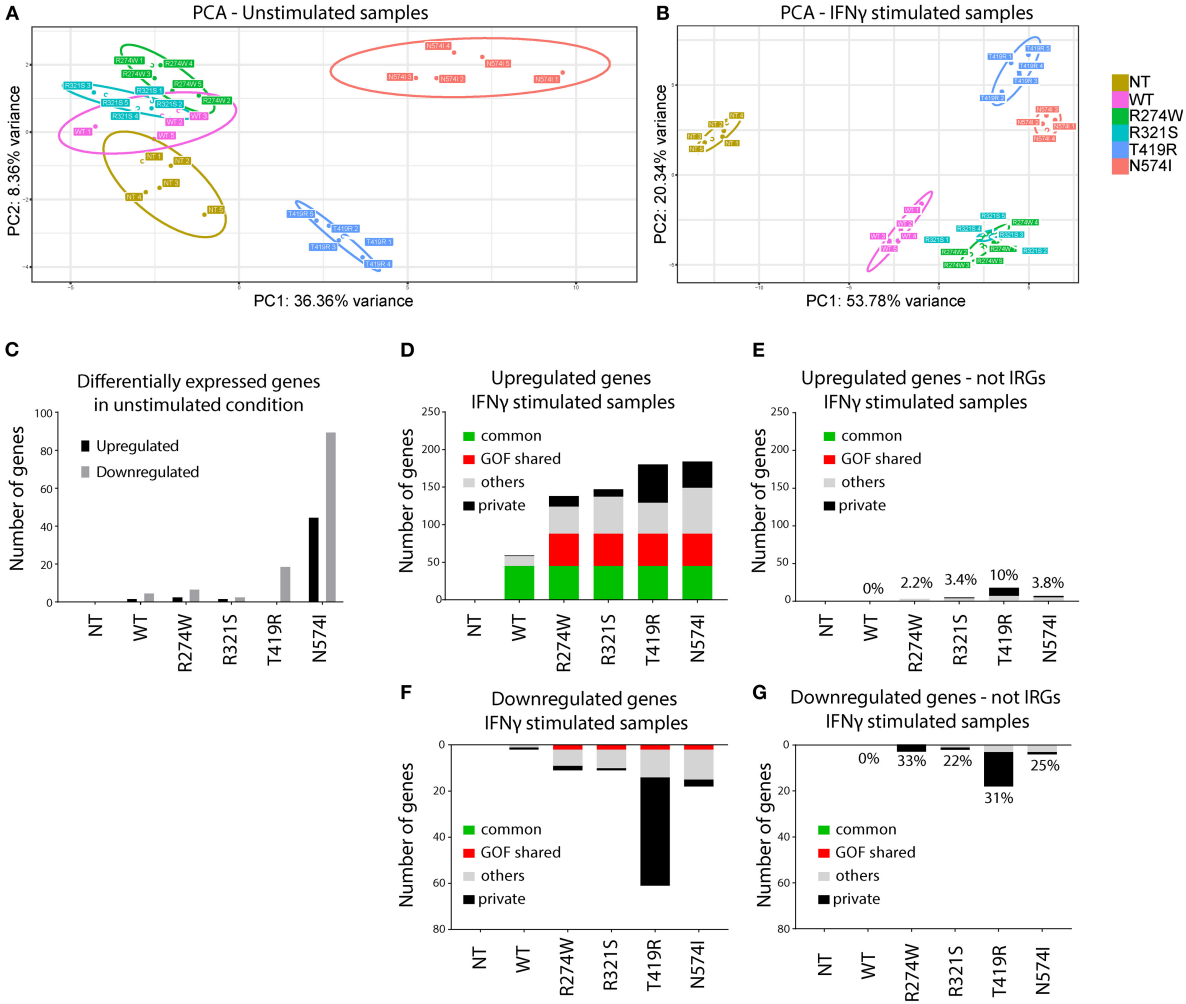
Principal component analysis (PCA) and number of de-regulated genes before and after IFNγ stimulation. **(A)** PCA of unstimulated samples. **(B)** PCA of IFNγ stimulated samples. Clustering of the different replicates per condition was observed and designated with different color codes. The colored circles indicate the 95% confidence ellipse for each cluster. **(C)** Number of genes differentially expressed in unstimulated samples compared with non-transduced U3A^−/−^ cells (NT). **(D)** Number of up-regulated genes (IFNγ stimulated condition was compared with the unstimulated condition for each condition). **(E)** Number of up-regulated genes present in **(D)** not previously reported as IRGs. On top of the bars, the percentage of non-IRGs among the total up-regulated genes. **(F)** Number of down-regulated genes (IFNγ stimulated condition was compared with the unstimulated condition for each condition). **(G)** Number of down-regulated genes present in **(F)** not previously reported as IRGs. On top of the bars, the percentage of non-IRGs among the total down-regulated genes. Indicated in green, genes that are commonly overexpressed by STAT1 WT and all GOF mutants. In red, genes overexpressed in all GOF mutants. In black, genes expressed only by that specific mutant. In gray, genes that are non-private or non-common WT or GOF. Here we plotted genes that showed at least 2-fold up- or down-regulation compared to their unstimulated condition (*p* < 0.01).

**Figure 3:**
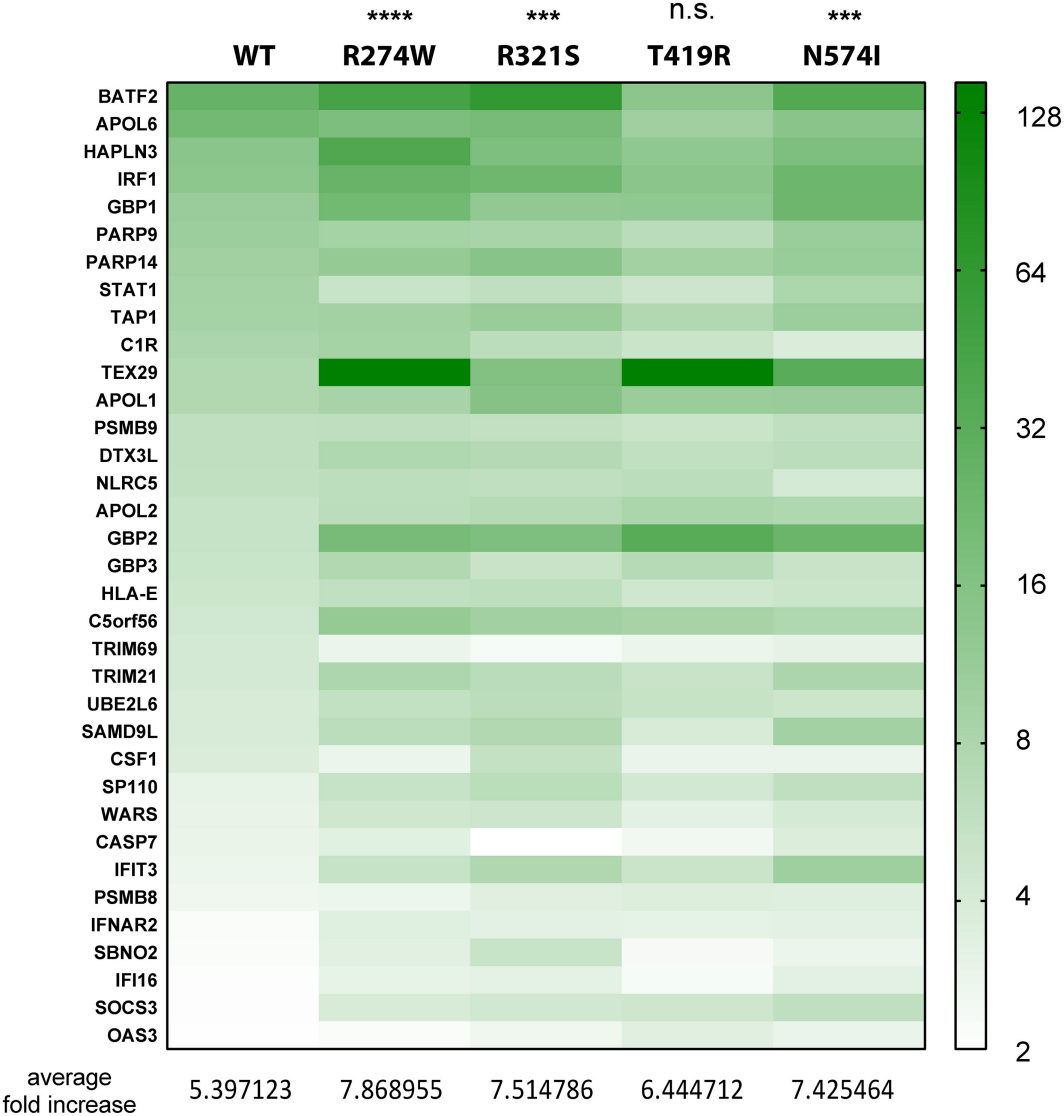
Heatmap indicating the expression level of genes commonly up-regulated in STAT1 WT and all GOF conditions, after IFNγ stimulation, presented in the green part in Figure 2D. Expression level is represented as fold increase compared to unstimulated condition as different shades of green (see scale bar at the right-hand side of the figure). Below the heatmap, the average fold expression increases are reported for the respective column. Statistical analysis indicated on top: Two-way ANOVA with Dunnett's multiple comparison test compared to WT stimulated condition: n.s. not significant; ***p < 0.001; ****p < 0.0001.

Two-way ANOVA with Dunnett's multiple comparison test was used to test the data in Figures 2, 3. Friedman test with Dunn's multiple comparison was used to compare the occurrence profile of the different mutants in **Figures 7A–C**. Pearson's Chi-squared test with Yates' continuity corrections was used to compare the proportions of ChIP-seq peaks in **Figures 7D,G**. ^*^p < 0.05, ^**^p < 0.01, ^***^p < 0.001, ^****^p < 0.0001 for all the figures.

## Results

### Experimental Setup

To study the transcriptomic fingerprints of the different STAT1 GOF mutations, we complemented a STAT1^−/−^ U3A cell line using lentiviral vectors to stably express STAT1-GFP WT or one of the respective STAT1 GOF mutations, R274W, R321S, T419R, or N574I, thereby generating isogenic add-back cell lines, referred to as STAT1 WT, R274W, R321S, T419R, and N574I. Non-transduced cells (hereafter termed NT) were taken along as control. To ensure comparable STAT1 expression levels, cells were generated using the highest dilutions of a limiting dilution series of lentiviral vectors and subsequent antibiotic selection of transduced cells, ensuring a polyclonal cell population with a single LV integration. Using a stable cell line, instead of transient transfection, allowed us to eliminate variability derived from different transfection efficiencies between experiments. Detailed characterization of these cell lines, comprising quantification of the respective proteins and nuclear translocation upon IFNγ stimulation by fluorescent microscopy and increased expression of three IFNγ regulated genes (IRGs: *IRF1, GBP1*, and *CXCL10*) was previously reported (24).

In an effort to study the transcriptomic fingerprint of STAT1 WT, the different GOF mutations, and non-transduced U3A STAT1^−/−^, each cell line was stimulated for 4 h with IFNγ or left unstimulated (*n* = 5 independent experiments per cell line). Total mRNA was extracted and sequenced on an Illumina HiSeq4000 (Methods). For each condition, we selected the genes showing at least a 2-fold difference between the unstimulated and the stimulated condition (*p* < 0.01, Benjamini-Hochberg procedure).

### PCA Reveals Distinct Clustering for the Different GOF Mutants

In a first step, RNAseq data were subjected to Principal Component Analysis (PCA), either for the unstimulated conditions or after IFNγ stimulation. This analysis reduces the dimensionality of the samples and clusters mRNA sequencing results based on their similarities over the main principal components. Samples with a similar transcriptomic profile will cluster closer together. PCA evaluates the whole transcriptomic fingerprint of the different conditions to define principal components (PC1 and PC2), and plots the respective replicates of the RNAseq experiment for each specific condition accordingly. PCA demonstrated clear clustering of the different replicates per condition and allowed to identify one of the five replicates of STAT1 WT in the unstimulated condition and one of N574I in the stimulated condition as outliers (Supplementary Figure 1). These were removed from subsequent analyses.

PCA of the remaining replicates under non-stimulated and stimulated condition is shown in Figures 2A,B, respectively. In the unstimulated condition (Figure 2A), STAT1 WT clusters closely to R274W and R321S, and to a lesser extend to NT, differing only for PC2 (8.55% of the variance). This suggested that addition of neither STAT1 WT nor these STAT1 GOF mutations alone significantly affected the transcriptome. On the other hand, N574I and T419R formed two distinct clusters, indicating a larger effect upon complementation with these two mutants even in the absence of IFNγ stimulation.

Upon IFNγ stimulation (Figure 2B), both STAT1 WT and all GOF mutants clustered away from the NT condition. Again, R274W and R321S mutant clusters overlapped and located close to the STAT1 WT cluster, while T419R and N574I formed two clusters separated from STAT1 WT and the other GOF mutants.

### T419R and N574I Have Already an Impact in Unstimulated Conditions

PCA showed that, even in unstimulated conditions, STAT1 T419R and N574I addition to U3A^−/−^ cells resulted in a shift relative to STAT1 WT. Therefore, we first determined the number of genes significantly (*p* < 0.01) up- or down-regulated (≥2-fold) relative to STAT1 WT, in unstimulated conditions (Figure 2C). As expected from the PCA analysis, R274W and R321S did not show major transcriptomic differences compared to STAT1 WT, with only one gene significantly up-regulated and five down-regulated for the R274W mutant and none differentially expressed for R321S mutant. On the contrary, both T419R and N574I mutants showed more transcriptomic differences under unstimulated conditions compared to STAT1 WT, with one up- and 21 genes down-regulated (on average 2.65-fold) for T419R and 44 genes (on average 2.96-fold) up- and 90 down-regulated (on average 2.70-fold) for N574I (Figure 2C).

Together this underscored that the stable introduction of STAT1 WT and the respective STAT1 GOF mutants, did not affect gene expression under basal conditions, although for T419R and N574I several genes were significantly down-regulated. Interestingly, none of the genes down-regulated by N574I and 3 out of 22 genes down-regulated by T419R in unstimulated conditions were up-regulated after IFNγ stimulation.

### STAT1 GOF Mutations Show a Wider Range of Up-Regulated IRGs

In a next step, we studied the impact of IFNγ stimulation on gene expression by first determining the number of up- and down-regulated genes for each condition relative to NT (Figures 2D–G). The NT condition did not show any significantly up- or down-regulated genes when comparing the conditions with and without IFNγ stimulation, confirming that absence of STAT1 completely abolished responsiveness to IFNγ (Figures 2D,F). Looking at the up-regulated genes, STAT1 WT as well as GOF showed a substantial set of up-regulated genes (Figure 2D): STAT1 WT showed fewer up-regulated genes (*n* = 59) than the GOF mutants (*n* = 138 for R274W, *n* = 147 for R321S, *n* = 180 for T419R, *n* = 184 for N574I). Sorting these up-regulated genes we identified 45 to be up-regulated by STAT1 WT and all the GOF mutants (referred to as commonly up-regulated; green bar, Figure 2D), and 43 genes exclusively up-regulated by all GOF mutants, but not by STAT1 WT (GOF shared; red bar). Of interest, only a single gene (*SMG1*) was up-regulated in STAT1 WT, but not in any of the GOF mutants, while a larger number of “private” up-regulated genes was observed for the GOF mutants (*n* = 1 for WT, *n* = 14 for R274W, *n* = 10 for R321S, *n* = 51 for T419R, *n* = 35 for N574I; private; black bar, Figure 2D). A smaller set of genes was up-regulated in two or more, but not all, GOF conditions (*n* = 13 for WT, *n* = 36 for R274W, *n* = 49 for R321S, *n* = 41 for T419R, *n* = 61 for N574I; mixed; gray bar, Figure 2D).

A small number of genes was down-regulated (Figure 2F) for STAT1 WT (*n* = 2, on average 2.7-fold), R274W (*n* = 11, on average 2.8-fold), R321S (*n* = 11, on average 2.6-fold) and N574I (*n* = 18, on average 2.7-fold), while T419R showed a larger number of genes down-regulated after IFNγ stimulation (*n* = 61, on average 2.6-fold).

The fact that a large set of genes was up-regulated in the GOF cells compared to STAT1 WT, led us to hypothesize that STAT1 GOF mutants may have lost specificity for IRGs. Therefore, we assessed how many dysregulated genes did not belong to the IFNγ pathway (using the Interferome.org, a manually curated database including 9,768 type II interferon genes) (Figures 2E,G). This analysis indicated that for the R274W and R321S mutants, only a small number of up- and down-regulated genes were non-IRGs (*n* = 3 up-regulated and *n* = 3 down-regulated for R274W, and *n* = 5 up-regulated and *n* = 2 down-regulated for R321S). For N574I, a larger set of genes up- and down-regulated were non-IRG (*n* = 7 up-regulated and *n* = 4 down-regulated). STAT1 T419R showed the largest difference by, respectively, up- and down-regulating 18 and 18 non-IRGs, respectively. The large majority of genes affected were up-regulated IRGs (Figures 2D,E), whereas a smaller number of genes was down-regulated (Figures 2F,G). Altogether, these results suggested a reduced specificity of the response to the IFNγ stimulus, resulting in more up-regulated IRGs in the GOF cells compared to WT.

### Overall Expression for Commonly Up-Regulated Genes Is Higher for STAT1 GOF

As a next step, we analyzed the relative expression levels of genes up-regulated by STAT1 WT and GOF mutants. First, we performed this analysis for the common up-regulated genes (green bar, Figure 2D) (Figure 3; expression heatmap). For the 35 common up-regulated genes, we calculated the average fold increase compared to the corresponding unstimulated lines: for the WT STAT1 condition an average 5.4-fold increase was obtained, whereas R274W, R321S, and N574I mutants resulted in a significantly higher fold increase (7.9, 7.5, and 7.4 average fold increase, respectively). T419R mutant did not result in a significant difference compared to STAT1 WT, with an average 6.4-fold increase in expression compared to unstimulated cells (*p* = 0.11, 2-way ANOVA compared to WT; Figure 3). These results highlighted that the genes up-regulated by STAT1 WT are also up-regulated by all STAT1 GOF mutants. Still, the up-regulation of these genes was significantly stronger for all GOF (overall 1.4-fold) compared to STAT1 WT, with the exception of T419R.

**Figure 4:**
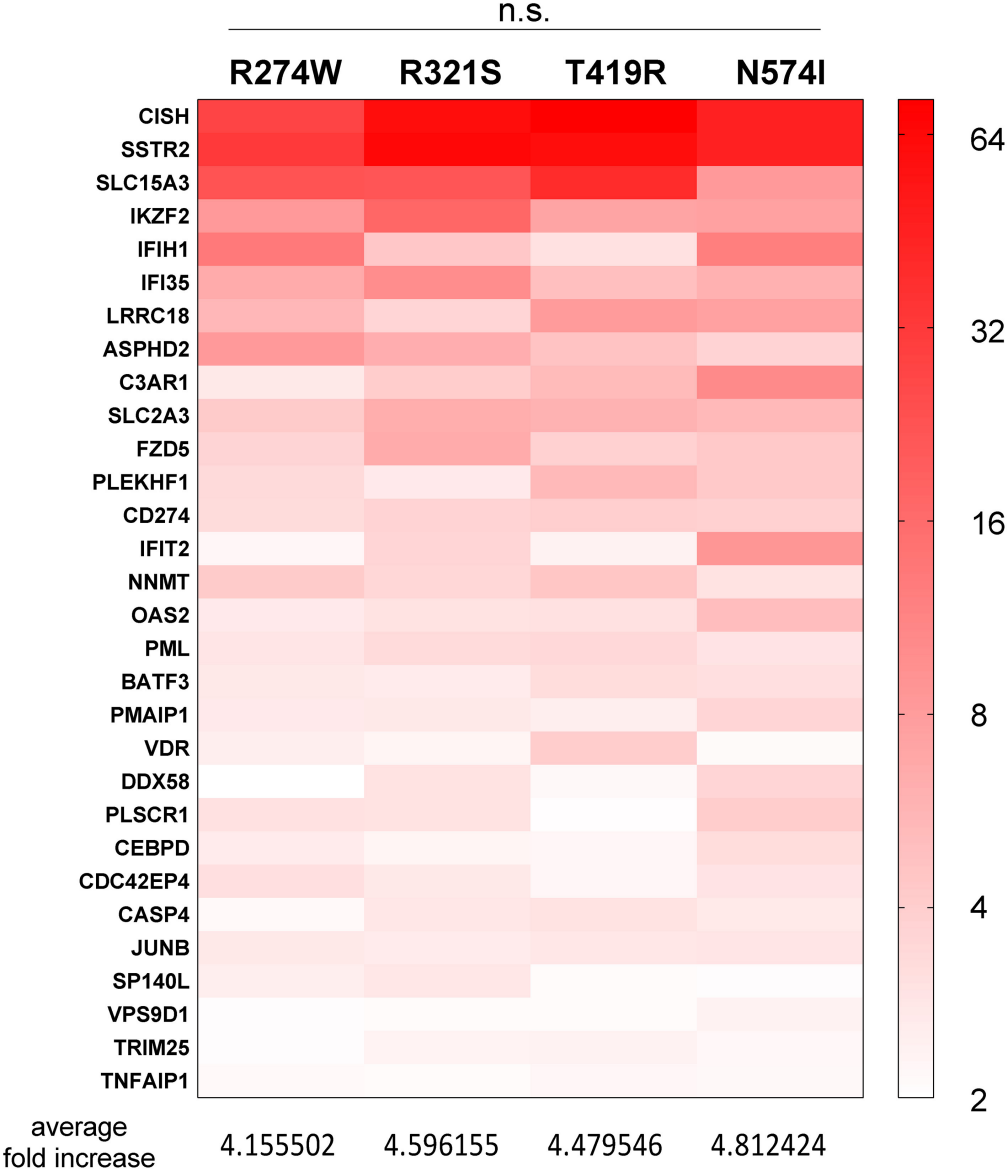
Heatmap representing the expression level of genes, present in the red part in Figure 2D, commonly up-regulated in all STAT1 GOF samples but not in STAT1 WT, after IFNγ stimulation. Expression level is represented as fold increase compared to unstimulated condition as different shades of red (see scale bar at the right-hand side of the figure). Below the heatmap, the respective average increases in fold expression are reported for the respective columns. Statistical analysis indicated on top: Two-way ANOVA with Dunnett's multiple comparison test, comparing each group to each other: n.s. not significant.

In addition, we identified genes that were up-regulated in all GOF conditions following IFNγ stimulation, but not in WT (red bar, Figure 2D). For these genes, we analyzed the average expression fold increase for the genes (Figure 4, red heatmap), which resulted in a 4.5-fold average increase for all GOF mutants (no significant difference, 2-way ANOVA). This result pointed at a subset of genes commonly up-regulated by GOF mutants, but not in WT conditions.

### Pathway Analysis Indicates Distinct Fingerprints of GOF Mutants

**Figure 5:**
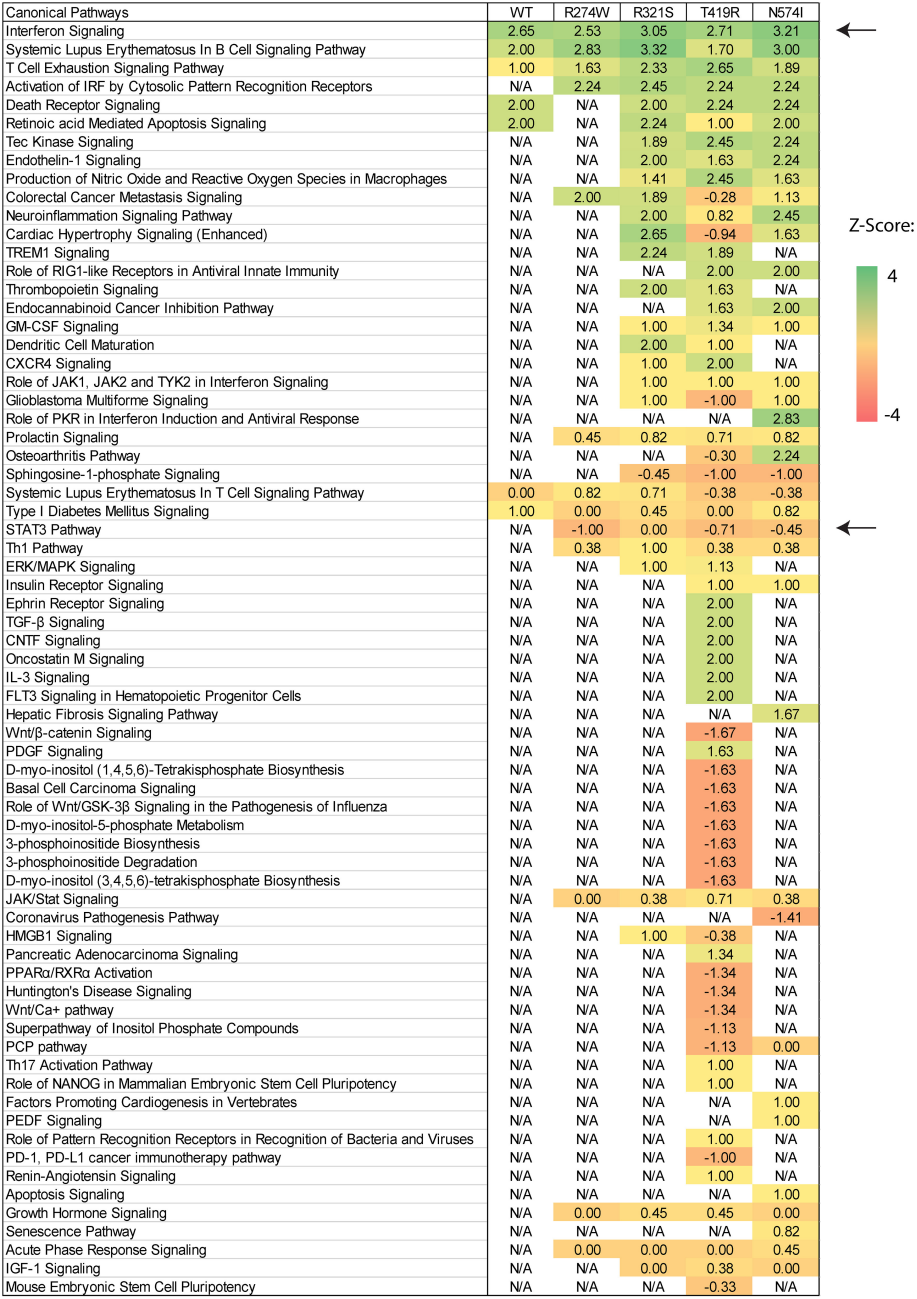
Canonical pathway prediction via IPA on differentially regulated genes for each condition after IFNγ stimulation. Pathway activation is scored using gene expression Z-scores and plotted in an activation Z-score matrix. Z-score represents a measure of how much the specific pathway is predicted to be up- or down-regulated (positive and negative score, respectively). Black arrows point to relevant pathway: Interferon signaling was up-regulated in all samples and STAT3 down-regulated in all GOF conditions, except for the R321S mutant (Z-score= −1, 0, −0.70, −0.47 for R274W, R321S, N574I, and T419R, respectively). For R321S the STAT3 pathway was significantly regulated yet without a clear predominance for up- or down-regulation.

After showing differences in the number of dysregulated genes and the magnitude of up-regulation, we investigated how these genes affect physiological pathways. The IPA software (Qiagen) was used to predict which pathways are up- or down-regulated by the different STAT1 WT or GOF mutants. In the unstimulated condition, comparing each sample to the NT sample, no significant pathway was predicted to be up- or down-regulated (result not shown). In contrast, comparing each condition before and after IFNγ stimulation, several pathways were predicted to be up- or down-regulated in both STAT1 WT and GOF mutants (Figure 5). The different pathways were sorted according to their activation Z-score (matrix of pathway activation scores, Figure 5). As expected, the highest Z-score was obtained for the interferon signaling pathway for both STAT1 WT and the different GOF mutants, with R321S and N574I showing the highest score amongst the GOF mutants. No significant Z-scores were detected when comparing IFNγ stimulated WT to IFNγ stimulated GOF samples (not shown).

**Figure 6:**
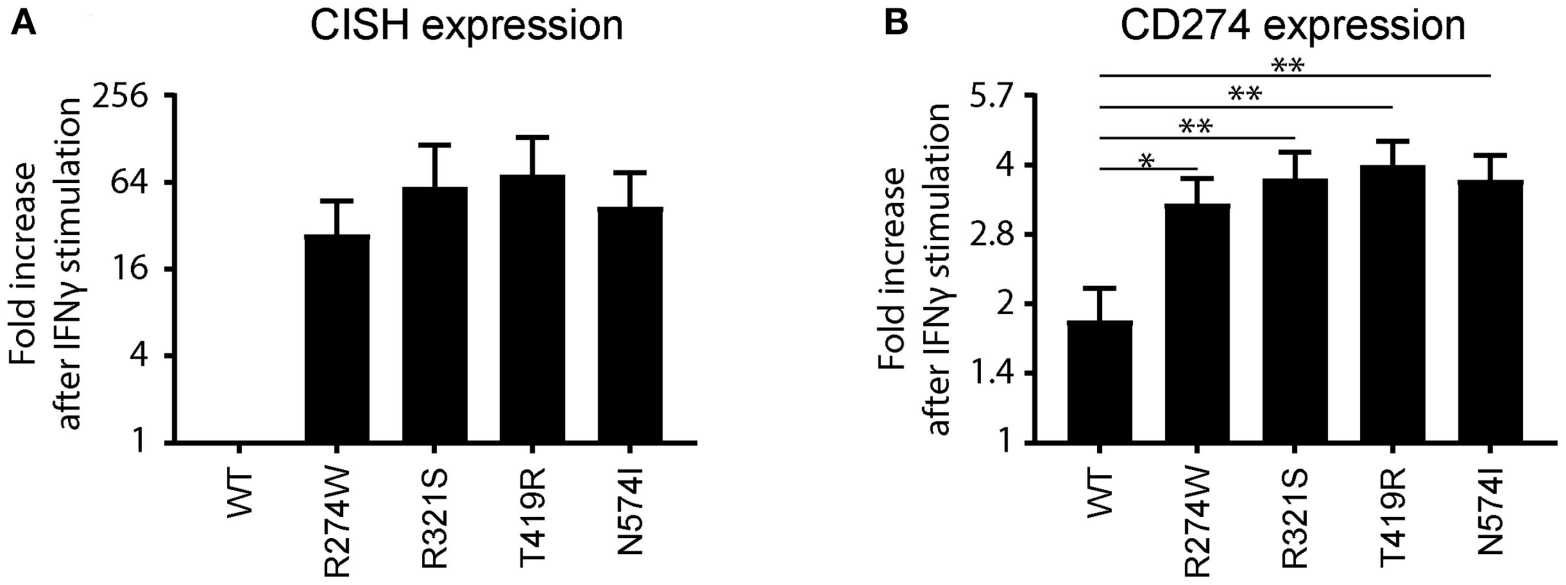
**(A)** *CISH* and **(B)** *CD274* (PD-L1) mRNA expression fold increase upon IFNγ stimulation relative to the non-stimulated condition for STAT1 WT or the respective GOF mutants represented as means ± standard errors. The fold increase for CISH in the IFNγ stimulated WT sample was found not to be significantly different from the non-stimulated STAT1 WT, and was therefore set to 1. Statistical analysis in **(B)** compares fold increase after IFNγ stimulation for each GOF sample to WT: one-way ANOVA with Dunnett's multiple comparison test compared to WT stimulated condition: *p < 0.05; **p < 0.01.

The R274W mutant showed limited differences in the number of up- or down-regulated pathways when compared to STAT1 WT. On the other hand, the T419R mutant showed the highest degree of difference from STAT1 WT profile (Figure 5). Importantly, T419R also showed down-regulation of different pathways that remained unaffected in both STAT1 WT and in the other GOF mutants. This result mirrored the observation that, upon IFN simulation, the T419R mutant down-regulates a higher number of genes compared to STAT1 WT than the other GOF mutants (Figures 2D,F). Of interest, IPA for R274W, T419R, and N574I showed that STAT3 signaling was down-regulated. In line with this, the *CISH* gene, a known inhibitor of STAT3 was strongly up-regulated upon IFNγ stimulation by all GOF mutants studied (>20-fold; Figure 6A). Together with the known link between STAT1 GOF and STAT3 pathway down-regulation (37), this could suggest that at least part of the commonly observed phenotype for GOF patients could be due to an inhibition of STAT3 signaling. In parallel, we observed a >2-fold up-regulation of *CD274* (encoding PD-L1) (Figure 6B) in all GOF samples, compared to their unstimulated condition (no IFNγ). When compared to WT, all STAT1 GOF mutants showed a significant *CD274* upregulation. Up-regulation of *CD274* was described to inhibit Th17 differentiation (38) and was shown to be present in patients affected by CMC after IL27 stimulation (39). The central role of Th17 cells in the pathogenesis of CMC, suggests involvement of *CD274* up-regulation, observed in the large majority of STAT1 GOF patients.

### STAT1 GOF Mutants Show a Loss of Specificity for the Canonical STAT1 Binding Motifs

Together, the previous analyses indicate that GOFs hyperactivate the commonly up-regulated genes compared to STAT1 WT, with the exception of T419R (Figure 3). Additionally, the STAT1 GOF mutants up-regulated a set of additional genes, part of which are common for all GOF studied here (Figure 2D, red bar), but a substantial part is unique or shared with at least one, but not all other GOF mutants. We hypothesized that this may be explained by the binding of STAT1 GOF to more diverse sequences than the canonical STAT1 consensus motif in IRG promoters.

We therefore identified and analyzed the promoters of the genes up-regulated after IFNγ stimulation. A short promoter region [from −200 to +50 bp around the Transcription Start Site (TSS)] was retrieved for all up-regulated genes using the Eukaryotic Promoter Database (EPD) web tool (40). As a control, we used promoter sequences from 100 randomly picked genes.

**Figure 7:**
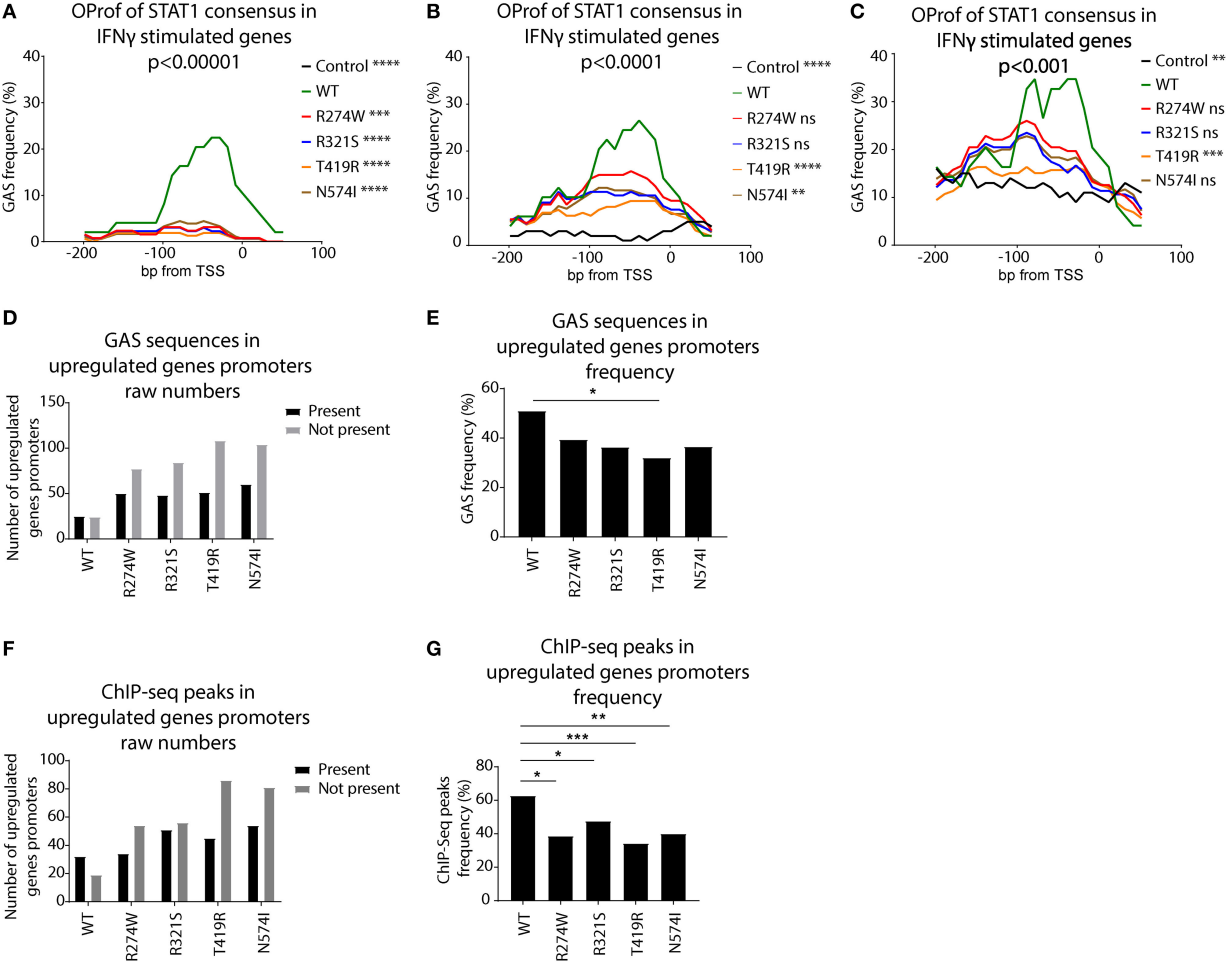
Occurrence profile (OProf) of IFNγ activation site (GAS) consensus sequences, predicted using the HOCOMOCO (34) GAS consensus at three different *p*-value thresholds for genes up-regulated upon IFNγ stimulation: **(A)** *p* < 0.00001, **(B)** *p* < 0.0001, and **(C)** *p* < 0.001. As control, we used promoter sequences from 100 randomly selected genes. Statistics **(A–C)**: Friedman test with Dunn's multiple comparison *post-hoc* test was performed comparing each group to STAT1 WT; ns not significant; **p < 0.01; ***p < 0.001; ****p < 0.0001. **(D)** Number of promoters containing GAS consensus occurrence in promoters of genes up-regulated upon IFNγ stimulation. Black bars show promoters containing at least one GAS consensus sequence, gray bars **(E)** Frequency of genes harboring at least one GAS consensus in their promoter over the total upregulated genes. **(F)** Number of promoters containing ChIP-Seq peaks for genes up-regulated upon IFNγ stimulation. Black bars represent promoters containing at least one ChIP-Seq peak. Gray bars show the number of promoters lacking a ChIP-Seq peak. **(G)** Frequency of genes harboring at least one ChIP-Seq peak in their promoter, over the total upregulated genes. Statistics **(D,E)**: Pearson's Chi-squared test with Yates' continuity correction; *p < 0.05; **p < 0.01; ***p < 0.001. OProf, occurrence profile; GAS, IFNγ activation site.

We scanned the promoters of these genes using a STAT1 GAS consensus Position Weight Matrix, with different levels of stringency (*p* < 0.001, *p* < 0.0001, and *p* < 0.00001). The frequency of predicted GAS consensus sequences for each promoter region of the up-regulated genes was plotted for these respective stringencies (Figures 7A–C). For STAT1 WT there was a clear 20–40% peak of GAS sequence frequency between the TSS and −100bp (green line, Figures 7A–C). Performing the same analysis for the promoters of genes exclusively up-regulated by STAT1 GOF mutants following IFNγ stimulation, no clear peaks were observed using the highest stringency parameters (*p* < 0.00001, Figure 7A). In a next step, we therefore reduced the stringency of the GAS consensus definition (*p* < 0.0001 and *p* < 0.001), allowing for more degenerate GAS sequences to be included. This resulted in an increased frequency of predicted GAS in the promoter region for STAT1 WT (green line, Figures 7B,C), as well as the GOF mutants, albeit not to the level of STAT1 WT, whereas frequencies in the control promoter set remained low (black line, Figure 7B). At a confidence level of *p* < 0.0001 (Figure 7B), the R274W and the R321S curves were no longer significantly different from STAT1 WT (Friedman test with Dunn's multiple comparison *post-hoc* test). Lowering the stringency further to *p* < 0.001 (Figure 7C) also resulted in the N574I not being significantly different from STAT1 WT. In contrast, the random control set and the T419R mutant remained significantly different from STAT1 WT.

Finally, we calculated the raw number of promoters (−200, +50 bp from TSS) of up-regulated genes harboring at least one canonical GAS consensus sequence and the ones without GAS consensus (Figures 7D,E). Whereas, for STAT1 WT, the number of up-regulated genes with and without GAS consensus was comparable (*n* = 25 and *n* = 24, respectively), there were more GOF stimulated genes that had promoters lacking a consensus GAS sequence (Figure 7D). We observed a reduction of the ratio GAS present over GAS not present for all the GOF mutants, reaching statistical significance only for T419R, however (Figure 7E, *p* = 0.025, Pearson's Chi-squared test with Yates' continuity correction).

Together, these results suggested that the promoters of STAT1 GOF up-regulated genes presented fewer canonical GAS motifs than WT STAT1 up-regulated genes, leading us to hypothesize that the STAT1 GOF mutants have reduced specificity for the canonical GAS consensus and activated a wider array of genes. In addition, in promoters of T419R up-regulated genes, GAS sequence frequencies remained very low, even when lowering the stringency of GAS consensus definition to *p* < 0.001 (Figure 7C, orange line). Interestingly, the T419R mutant was the only one not significantly different from the control group at both *p* < 0.001 and *p* < 0.0001 stringency levels and harbored fewer canonical GAS motifs in the promoters of its up-regulated genes.

### STAT1 ChIP-Seq Peaks Suggests Altered GAS Consensus Recognition

The previous analysis showed that the STAT1 GOF mutants recognized the canonical GAS sequences (the genes up-regulated by STAT1 WT were also up-regulated by STAT1 GOF) as well as more degenerated GAS sequences. Interestingly, Robertson et al. (35) performed ChIP-Seq to identify mammalian DNA target sequences bound by endogenous STAT1 in unstimulated and IFNγ-stimulated HeLa-S3 cells (30 min). This dataset compiled endogenous STAT1 binding sites and contains >18.000 peaks. We used these ChIP-Seq data as a proxy for STAT1 GAS sequences, and determined the frequency of ChIP-Seq reads for all promoter sequences of the genes up-regulated in STAT1 WT and GOF cell lines (Figure 7F). For each STAT1 cell line, we determined which of the respective promoters harbored a ChIP-Seq peak and plotted the ratio between the number of genes harboring at least one ChIP-Seq peak in their promoter, over the number of genes not harboring any ChIP-Seq peak. For the GOF mutants, we analyzed only the genes not already up-regulated by STAT1 WT, to highlight the GOF-specific impact on GAS recognition. All GOF mutants showed a higher proportion of promoters lacking a ChIP-Seq peak, compared to STAT1 WT, suggesting that the promoter of these genes under standard conditions does not get frequented by STAT1 upon IFNγ stimulation. As these genes are nonetheless up-regulated upon IFNγ stimulation, STAT1 GOF mutants likely recognize more divergent, non-canonical GAS sequences. For STAT1 R274W and R321S the differences with STAT1 WT were less pronounced, suggesting that these two mutants may be less promiscuous.

## Discussion

To date, 105 different mutations in STAT1 have been associated with a GOF and more are identified each year. Most STAT1 GOF patients are affected by CMC, but a wider range of phenotypes has been associated with this disease. Symptomatic treatment has been inconsistent, with varying response to jakinibs (22, 41–43). It remains unknown whether this clinical heterogeneity can, at least in part, be explained by differences in STAT1 GOF molecular mechanisms or whether this is mainly driven by the genetic background of the patient.

Recently, we demonstrated that distinct dynamics can underlie STAT1 GOF for a subset of STAT1 GOF mutations that spread over the protein and associate with diverse GOF phenotypes in patients (STAT1 GOF R274W, R321S, T419R, and N574I), including faster nuclear accumulation and reduced mobility in the nucleus following IFNγ stimulation (24). These results were in line with other reports (21–23).

In the present study, we hypothesized that the different molecular mechanisms identified for various GOF mutants may also affect the transcriptomic fingerprint of each mutant, in an effort to shed light on a potential link between molecular mechanism and varying phenotypes.

We performed mRNA sequencing in an isogenic U3A *STAT1*^−/−^ cell model complemented with STAT1 wild type or STAT1 GOF R274W, R321S, T419R, and N574I and compared their transcriptomes, with and without IFNγ stimulation, to identify dysregulated pathways extending beyond the group of immediate IFNγ-response genes.

Our study highlights that STAT1 GOF mutants (with the exception of T419R) up-regulated a subset of genes upon IFNγ that is common to STAT1 WT. In addition, another subset of genes up-regulated (such as *CISH, CD274*) was shared by all the GOF mutants, possibly associated with the common GOF phenotype. Finally, each mutant also presented a private subset of up- and down-regulated genes, possibly associated with mutation specific differences. Our analysis indicated that loss of specificity for the canonical GAS consensus sequences might drive this broader activation pattern. The most outspoken effect was observed for the T419R mutant, which up- and down-regulated multiple non-IRGs. We hypothesize that this is a result of the altered contact with genomic DNA.

The demonstration of distinct transcriptomic fingerprints for specific STAT1 GOF mutants upon IFNγ stimulation, may be explained by differences in the molecular mode of action of the respective GOF mutations, described previously by our group and others (21–24).

To our knowledge, this is the first study comparing the effect of different STAT1 GOFs on gene expression in an isogenic cell model. In 2017, Fujiki et al. (21) performed mRNA sequencing of STAT1 R274Q mutant in U3C cells. Similar to our results, the authors observed an overlap of genes modulated by both STAT1 WT and the R274Q mutant. In contrast to our results, they did not observe a significant difference in expression magnitude between STAT1 WT and R274Q. This discrepancy may be attributed to the different conditions used: 24 h of IFNγ stimulation using 2 biological replicates compared to 4 h of IFNγ stimulation using 5 biological repeats in our study. We specifically chose the 4 h time-point in order to monitor mRNA status early after IFNγ addition: we wanted to study the direct effect of the STAT1 GOF mutations on gene transcription, and aimed to limit the activation of secondary genes/pathways. Additional time points would be valuable to study. Still, in line with our results, they showed a similar TSS-proximal ChIP-Seq peak distribution at IFNγ modulated genes for STAT1 WT and R274Q mutant. In 2018, Ovadia et al. (26) described a patient with a H629Y STAT1 GOF mutation, located in the STAT1 SH2 domain. RNA-seq analysis on U3A cells transiently transfected with STAT1 WT or H629Y, and R274G STAT1 GOF mutants and revealed a minority of common GOF IFNγ-induced genes and a larger amount of mutation-specific up-regulated genes. In line with this, Meesilpavikkai et al. (39) described a V653I STAT1 GOF mutation, also residing in the SH2 domain resulting in a common STAT1 GOF phenotype (CMC and autoimmunity), together with atypical diverse infections and impaired cytokine regulation. This might allude to a combination of dysregulation of a common trunk of GOF genes together with mutation-specific dysregulations.

Our study has several limitations. First, we leveraged a U3A cancer cell line, depleted for endogenous STAT1 and complemented with STAT1 WT or GOF mutants using lentiviral vector expression. This resulted in cell lines homozygous for the GOF mutations, in contrast to their heterozygous context in patients. Second, the use of a cancer cell line could affect the transcriptomic fingerprint and limit extrapolation toward patient-derived cells and to the patients' phenotype. Indeed, this study does not explain the phenotypic variation observed between STAT1 GOF patients, and additional experiments in immunologically more relevant cells are required to verify this in more detail. However, complementing a *STAT1*^−/−^ cell line with the respective GOF mutations, allowed us to evaluate the mutations in an isogenic setting and to address additional confounders, with the only difference between each of our groups being a single point mutation in STAT1, unaffected by donor-to-donor variability. Moreover, the limited number of patients that can be sampled per STAT1 mutation (105 different mutants for over 400 patients described), currently complicates genotype-phenotype association studies, especially for the more divergent clinical presentations and even more rare associated features (such as vascular abnormalities). We believe that binning these STAT1 GOF mutations into mechanistically homogenous groups might help organize the observed phenotypic heterogeneity. In our work, each GOF mutant up- and/or down-regulated a subset of private genes, possibly associated with mutation-specific phenotypes, although pathway analysis did not show a clear correlation. Third, the STAT1 ChIP-Seq analysis derived from HeLa-S3 cells, was used to evaluate data obtained in U3A cells, similarly to Fujiki et al. (21), possibly affecting the correlation. Nevertheless, the IFNγ-stimulated STAT1 WT condition demonstrated a high proportion of ChIP-Seq peaks in stimulated genes, indicating an important degree of concordance between these models. We analyzed small TSS-proximal regions of the up- or down-regulated genes (−200 to +50 bp), to focus on the regulatory sequences strictly correlated with these genes, although regulatory regions located further upstream have been implicated in regulation by STAT1 (44, 45). Finally, we studied only the effect of IFNγ, and therefore STAT1 homodimerization, on the transcriptome. STAT1 is known to also form heterodimers with STAT2 upon IFNα stimulation, and STAT1/STAT2 heterodimers may also be involved in the STAT1 GOF pathogenesis. Likewise, STAT1/STAT3 heterodimers can be formed upon IL6 or IL27 stimulation (46). Neither of these heterodimers were addressed in this study. The effect of the different STAT1 GOF mutants on the transcriptome upon type I IFN, or IL6/IL27 stimulation would therefore certainly be worth analyzing.

In conclusion, our work further expands the knowledge on STAT1 and STAT1 GOF mutations. Our data are in line with previous findings of different molecular routes toward a STAT1 GOF, but also provide evidence for common and GOF mutation-specific impacts on the transcriptome, which in part may explain STAT1 GOF phenotypic presentation, but this requires additional experiments in physiologically more relevant cells. In-depth analysis of gene expression patterns associated with STAT1 GOF mutations enhances our understanding of the complexity of STAT1 GOF pathophysiology and genotype-phenotype correlations. In addition, the identification of specific STAT1 GOF up-regulated genes, can improve our prognostic capacity to identify and possibly stratify patients affected by this disorder, and may ultimately open up new avenues for disease interrogation and targeting.

Our work indicates how some STAT1 GOF mutants have lost specificity for canonical GAS sequences. All GOF mutants up-regulated genes for which a higher proportion of promoters lacked a ChIP-Seq peak compared to STAT1 WT, suggesting that the promoter of these genes is normally not bound by STAT1 upon IFNγ stimulation. The fact that their gene expression is up-regulated in STAT1 GOF underscored that STAT1 GOF recognized non-canonical GAS sequences as well. For the mutant affecting a DNA-interacting aa, T419R, even promoters lacking GAS sequences showed IFNγ-mediated stimulation. Most importantly, this work revealed how single point mutations, even when resulting in a similar common phenotype, may affect the transcriptome differently, helping to explain the pathogenesis of the wide range of STAT1 GOF phenotypes.

## Data Availability Statement

The datasets presented in the study were submitted to GEO database. This data can be found here: https://www.ncbi.nlm.nih.gov/geo/query/acc.cgi?acc=GSE166392.

## Author's Note

While the first STAT1 gain-of-function (GOF) mutations were described in 2011, a mechanistic correlation between the patients' mutations and their phenotype is still missing. The major cellular hallmarks of this disorder is increased phosphorylation of STAT1 protein and increased expression of interferon regulated genes. In this study, we analyzed different STAT1 GOF in an isogenic U3A-cell model to correlate the transcriptome with the underlying molecular mechanism, previously characterized by our group. We show how STAT1 GOF hyper-activate the physiological STAT1-regulated genes and on top dysregulate an additional set of GOF shared and mutation-specific genes upon interferon gamma (IFNγ) stimulus, including CISH and CD274, two genes involved in the inhibition of the STAT3 pathway and Th-17 differentiation. Following analysis of GAS sequence occurrence in the respective promoter regions, we observed different degree of loss of specificity for the canonical STAT1 consensus GAS sequence by STAT1 GOF mutants, depending on the specific GOF mutation. Overall, this work contributes to define STAT1 GOF mutations as a heterogeneous group of mutations, sharing common features and, at the same time, different underlying molecular mechanisms and degrees of transcriptional dysregulation.

## Author Contributions

SG carried out the experiments and analyzed the data. JD provided help and expertise for the analysis. RG and RS supervised the project. SG, RG, and RS conceived the study, interpreted the data and wrote the manuscript with input from all authors. All authors contributed to the interpretation of the results.

## Conflict of Interest

The authors declare that the research was conducted in the absence of any commercial or financial relationships that could be construed as a potential conflict of interest.
