# Supplementary material for: Transcriptional Profiling of STAT1 Gain-of-Function Reveals Common and Mutation-Specific Fingerprints
Source: Front Immunol. 2021 Feb 17;12:632997. doi: 10.3389/fimmu.2021.632997 (PMC7925617; doi:10.3389/fimmu.2021.632997)
Supplement: Supplementary file 1 [file Data_Sheet_1.pdf]

Supplementary Figure 1:

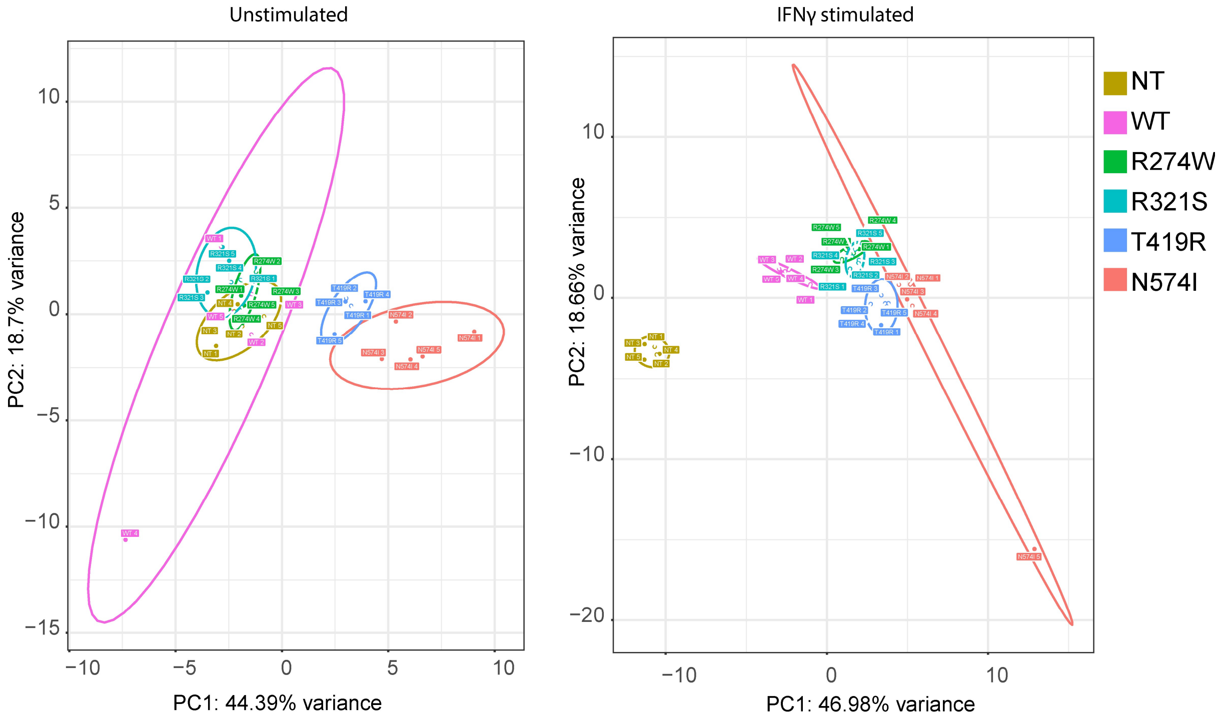

Supplementary Figure 1: Principal component analysis in unstimulated and IFNγ stimulated conditions. Two outliers (in unstimulated WT condition 4, and in IFNγ stimulated N574I condition 5) were identified and omitted from all further analyses.
